# Disease pathways at the Rat Genome Database Pathway Portal: genes in context—a network approach to understanding the molecular mechanisms of disease

**DOI:** 10.1186/s40246-014-0017-8

**Published:** 2014-09-30

**Authors:** Victoria Petri, G Thomas Hayman, Marek Tutaj, Jennifer R Smith, Stanley JF Laulederkind, Shur-Jen Wang, Rajni Nigam, Jeff De Pons, Mary Shimoyama, Melinda R Dwinell, Elizabeth A Worthey, Howard J Jacob

**Affiliations:** 1 Human and Molecular Genetics Center, Medical College of Wisconsin, Milwaukee, WI, USA; 2 Department of Physiology, Medical College of Wisconsin, Milwaukee, WI, USA; 3 Department of Pediatrics, Medical College of Wisconsin, Milwaukee, WI, USA; 4 Department of Surgery, Medical College of Wisconsin, Milwaukee, WI, USA

**Keywords:** Molecular pathway, Disease pathway, Altered pathway, Ontology, Systems biology

## Abstract

**Background:**

Biological systems are exquisitely poised to respond and adjust to challenges, including damage. However, sustained damage can overcome the ability of the system to adjust and result in a disease phenotype, its underpinnings many times elusive. Unraveling the molecular mechanisms of systems biology, of how and why it falters, is essential for delineating the details of the path(s) leading to the diseased state and for designing strategies to revert its progression. An important aspect of this process is not only to define the function of a gene but to identify the context within which gene functions act. It is within the network, or pathway context, that the function of a gene fulfills its ultimate biological role. Resolving the extent to which defective function(s) affect the proceedings of pathway(s) and how altered pathways merge into overpowering the system’s defense machinery are key to understanding the molecular aspects of disease and envisioning ways to counteract it. A network-centric approach to diseases is increasingly being considered in current research. It also underlies the deployment of disease pathways at the Rat Genome Database Pathway Portal. The portal is presented with an emphasis on disease and altered pathways, associated drug pathways, pathway suites, and suite networks.

**Results:**

The Pathway Portal at the Rat Genome Database (RGD) provides an ever-increasing collection of interactive pathway diagrams and associated annotations for metabolic, signaling, regulatory, and drug pathways, including disease and altered pathways. A disease pathway is viewed from the perspective of networks whose alterations are manifested in the affected phenotype. The Pathway Ontology (PW), built and maintained at RGD, facilitates the annotations of genes, the deployment of pathway diagrams, and provides an overall navigational tool. Pathways that revolve around a common concept and are globally connected are presented within pathway suites; a suite network combines two or more pathway suites.

**Conclusions:**

The Pathway Portal is a rich resource that offers a range of pathway data and visualization, including disease pathways and related pathway suites. Viewing a disease pathway from the perspective of underlying altered pathways is an aid for dissecting the molecular mechanisms of disease.

## 1 Background

### 1.1 Pathways and the Pathway Ontology

**Figure 1:**
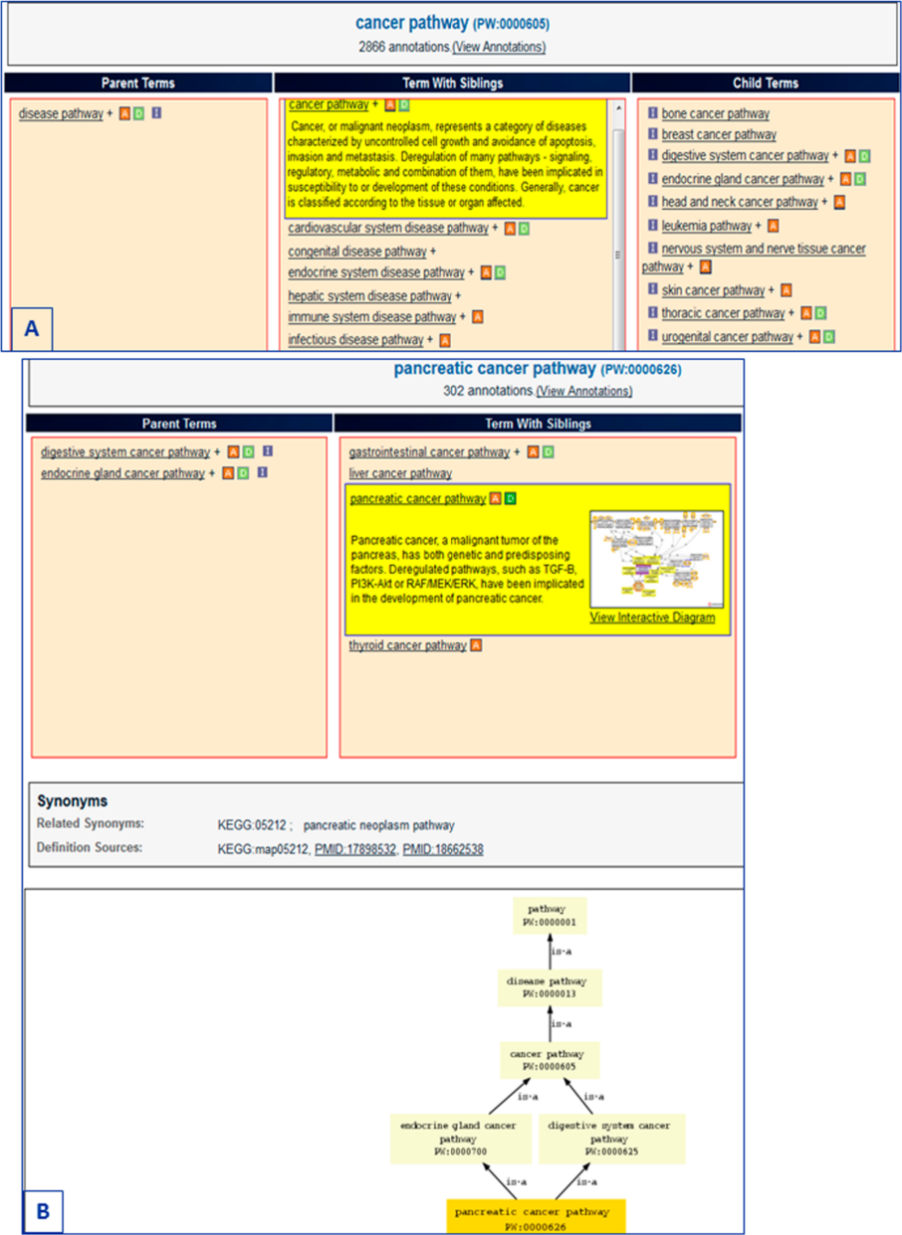
**The Pathway Ontology disease node and entries examples. (A)** The disease node and entries within. **(B)** The ‘pancreatic cancer pathway’ entry and position within the ontology.

Molecular networks or pathways represent sets of interactions and reactions that together form a functional unit. While the physical boundaries of such units are in many instances fluid, their delineation by the research community establishes a paradigm for studying the working of biomolecules within a context. These can be the sets of reactions and interactions that underlie the transformation of compounds, those that are initiated or triggered by a signaling event, or those that aim to maintain the normal status quo of cells and tissue. As such, they define the metabolic, signaling, and regulatory pathways, respectively. However, when one or more components within such sets are deviant, their effects can lead to an overall disturbance of the system. This is the realm of altered pathways whose combined effects may merge into the diseased state—the disease pathway. Finally, there are the sets of reactions and interactions that illustrate the responses of the system to the treatment intended to handle the diseased state. They constitute the drug pathways that include the pharmacokinetics and pharmacodynamics drug pathways—how the system processes/metabolizes the drug and how the drug affects the system, the drug-target interaction(s) and response(s), respectively. Drugs and other compounds can elicit adverse effects, at times severe enough to be toxic; such reactions offer a toxicogenomic perspective and are viewed as regulatory response networks. The various categories/types of pathways are organized within a hierarchical structure in the Pathway Ontology (PW) [1], developed and maintained at the Rat Genome Database (RGD) [2],[3]. The five nodes of the ontology are metabolic, regulatory, signaling, disease, and drug pathways. As necessary, terms for the altered version(s) of pathways are provided. The ontology allows for the standardized annotation of rat, human, and mouse genes to pathway terms. It also enables the deployment of interactive pathway diagram pages and allows for links between diagrams, ontology, and gene report pages, between connected pathways within diagrams, and between related pathways in pathway suites and suite networks. As such, it is the essential navigational tool of the Pathway Portal and, overall, within RGD [4],[5]. PW has also been instrumental for the building of pipelines to bring in pathway annotations done by other groups: the Pathway Interaction Database (PID) [6] pipeline for human gene annotations, extended to the rat and mouse orthologs, and the Kyoto Encyclopedia of Genes and Genomes (KEGG) [7],[8] pipeline, for rat, mouse, and human gene annotations. Pathway entries, as available at the sites, were mapped to PW terms as synonyms, and the genes associated with them were matched to RGD rat, human, or mouse genes using the Entrez gene ID and symbol [1]. Currently, these two pipelines are no longer running as PID has since retired and KEGG has changed its license; however, the annotations, as last brought in, are still available. Building the two pipelines benefited the ongoing development of the ontology, as more terms were added in order to provide for the mapping. In particular, the disease pathway node saw a substantial increase through the addition of terms for infectious diseases, substance abuse, and immune system diseases. More pipelines are planned to be built in the future. The cancer pathway entry and the pancreatic cancer pathway entry in the disease pathway node are shown in Figure 1A, B. In the ontology browser, A stands for annotations, D stands for diagrams, and the light versus dark shade(s) distinguish between annotations and/or diagrams to children or to term(s), respectively. A plus sign in front of a term designates the presence of children for that term.

### 1.2 Disease and altered pathways—a network approach to diseases

A disease pathway, as opposed to the clinical, gene-centric view of the condition, offers a network-centric view—an approach increasingly being considered by scientists. Interestingly, despite a wealth of information on mutated genes, the number of those considered ‘driver’ genes in cancer (genes that can promote or ‘drive’ tumorigenesis) is relatively small. Collectively, they are found in a set of important pathways such as the Ras-driven Erk1/2 and phosphatidylinositol 3-kinase-Akt signaling, those mediated by receptor tyrosine kinases (RTK), or those that are involved in cell cycle and apoptosis, DNA damage control, chromatin modification, and transcriptional regulation [9],[10]. Although the rest of the mutant genes are considered ‘passengers’, their presence and faulty interactions could, at least in principle, augment in some fashion the negative impact of ‘driver’ genes and, as such, contribute to the altered pathways leading to disease. Given the role these pathways normally play, their alterations can easily fuel the ‘hallmarks’ of cancer proposed by Hanahan and Weinberg: sustaining proliferative signaling, evading growth suppressors, activating invasion and metastasis, enabling replicative immortality, inducing angiogenesis, and resisting cell death [11]. In addition to variant genes and their associated dysfunctional pathways, deregulations stemming from sources other than protein coding genes such as those associated with microRNAs (miRNAs) and other non-coding elements or from sources other than sequence variation such as those at the epigenetic level can and do contribute to the disease phenotype. Abnormal chromatin modification as well as the up- and down-regulated expression of miRNA in tumor tissues compared to normal ones have been reported [10],[12]. Systems level, high-throughput, and -omics approaches to the study and understanding of disease initiation and progression are being considered [13],[14]. Disease pathways are approached in a similar, network-centric vein at RGD, and they are presented as sets/collections of altered pathways.

**Figure 2:**
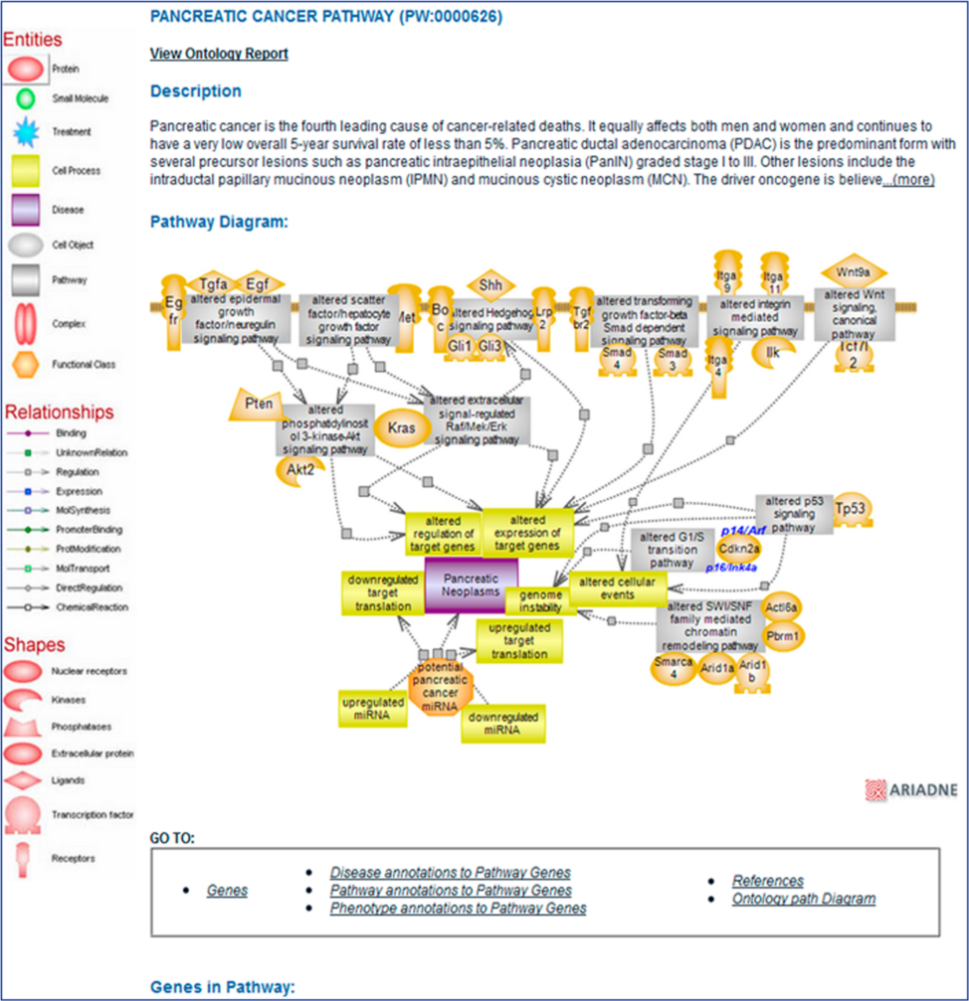
**Pancreatic cancer pathway diagram page.** The interactive pathway diagram page for the ‘pancreatic cancer pathway’. The altered pathways implicated in the condition are shown as gray rectangles, and they link to their respective ontology report pages. Culprit genes within each pathway are shown color-coded (the default color is red). The icon for the microRNAs (miRNAs) potentially implicated in the condition links to a page where several miRNAs are listed with additional links and references. The disease icon links to the Cancer Disease Portal in RGD.

**Figure 3:**
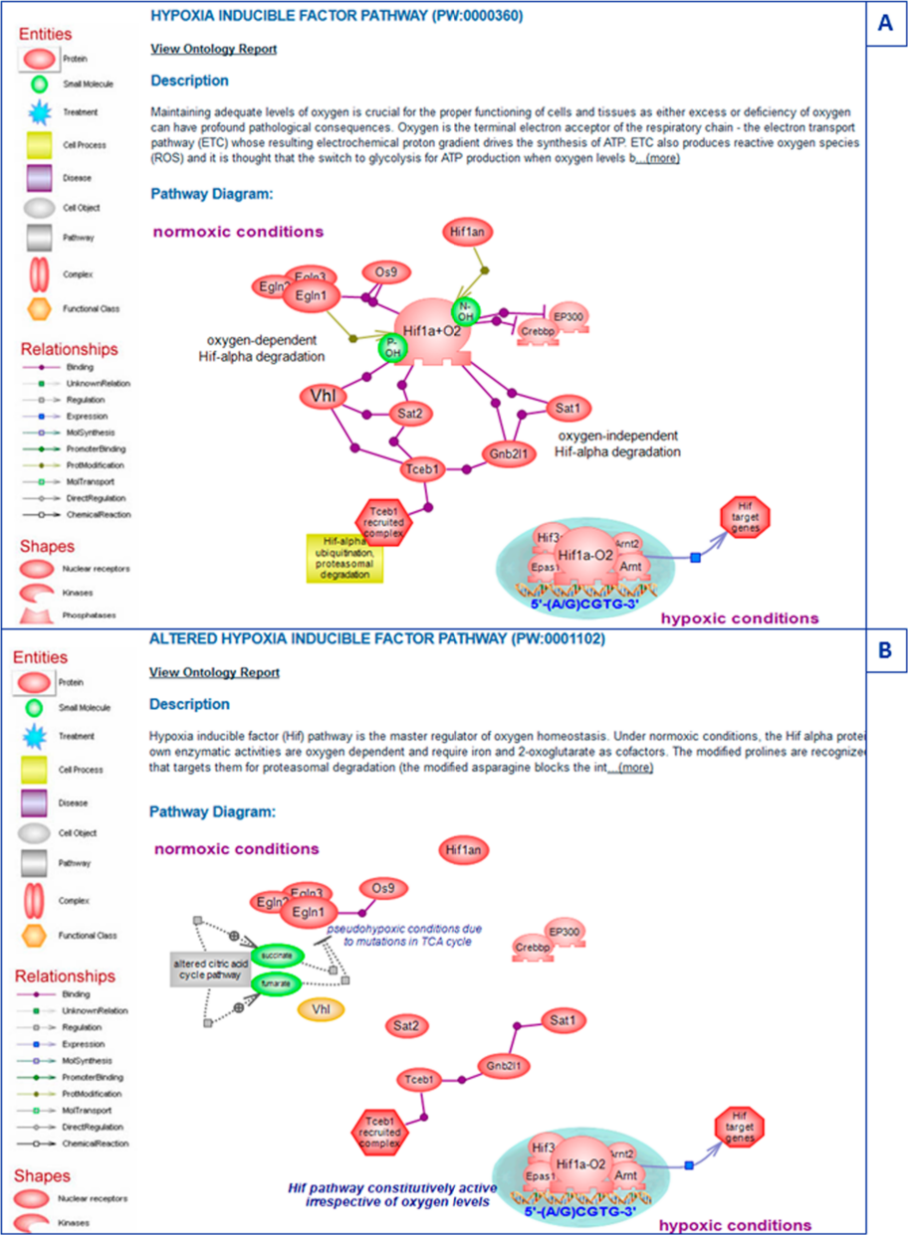
**Hypoxia-inducible factor pathway. (A)** The regular ‘hypoxia inducible factor pathway’. **(B)** The ‘altered hypoxia inducible factor pathway’.

**Figure 4:**
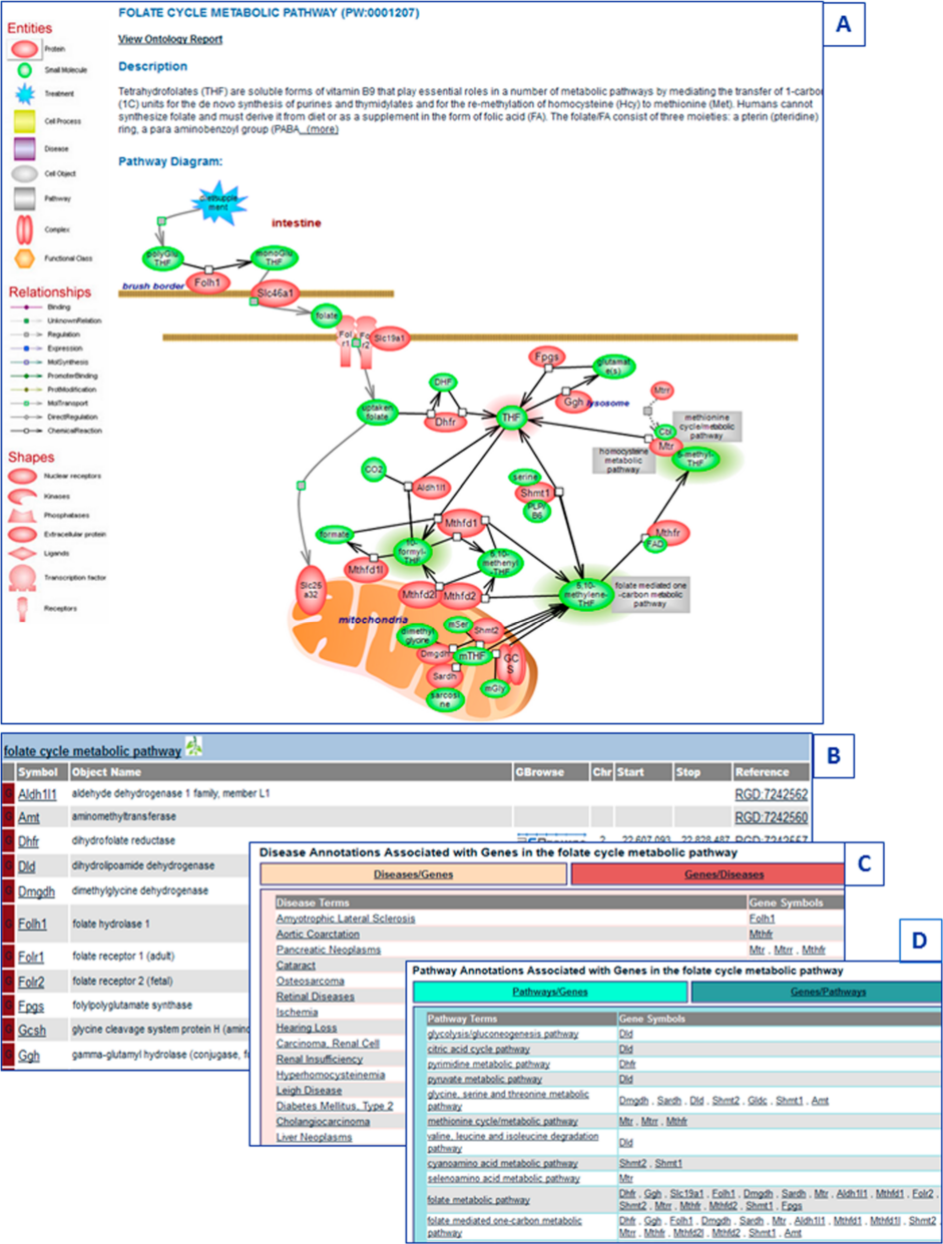
**The elements of an interactive pathway diagram page using the folate cycle metabolic pathway as an example. (A)** The synopsis of the pathway is shown at the top of the page with the option of viewing the entire text; the diagram of the pathway is below the description. **(B)** The genes in the pathway are shown by species in tabular form and there are various link options. **(C)** Genes in the pathway that have disease annotations are shown in tabular form that can be toggled between the alphabetically listed diseases with the associated genes shown to the right (default) and the alphabetically listed genes with the associated diseases shown to the right. **(D)** Genes in the pathway that have pathway annotations are shown in tabular form that can be toggled between the alphabetically listed pathways with the associated genes shown to the right (default) and the alphabetically listed genes with the associated pathways shown to the right. The last section of the diagram page contains the reference list and a view of the ontology tree (not shown).

The provision of terms for altered pathways and the representation of disease pathways and diagrams as collections of altered pathways are features unique to PW and its use at RGD [1],[4],[5]. The organization of individual disease pathway terms follows the structure of disease vocabularies with respect to the type of disease, organ, or tissue location, such that navigating this node can be both easy and meaningful to the user [see Figure 1]. Within an interactive diagram page for a disease pathway, those pathways known to be altered in the condition are represented with the culprit genes color-coded. As an example, the pancreatic cancer pathway is shown in Figure 2. Also color-coded are the culprit genes in a diagram for the altered version of the pathway [Figure 3B]. Connections or relationships between elements in the altered pathway are removed and/or changed, as appropriate, compared to the normal pathway proceedings [compare Figure 3A, B]. Any connected pathway shown on a diagram as a gray rectangle [see for instance Figures 2 and 3B] links to its ontology report and, from there, to a diagram, if one exists. Every gene entry links to its gene report page. Other links are provided for entries that group together several components/elements such as complexes, target genes, differentially expressed miRNAs, or other candidate genes. These are pages created within a content management system (CMS), and the individual elements in a page have links to corresponding report and/or other pages [see for instance ‘potential pancreatic cancer miRNA’ in Figure 2 and ‘Tceb1 recruited complex’ or ‘Hif target genes’ in Figure 3]. Links to drug or chemical compound ontology reports or sites or to pertinent disease portals at RGD are provided, as applicable. Every diagram page contains a synopsis with various links, and in the case of an altered pathway, a direct link to its normal version. The diagram page contains the list of genes in the pathway—by species (rat, human, mouse) or all, and tabular lists for genes in the pathway that have annotations to disease, pathway, and/or phenotype terms (the lists can be toggled). Finally, each page contains references that link to the PubMed abstract entry and a graph view of the path(s) to the term in the ontology [Figure 4] (graph not shown in Figure 4). PW is in the form of a directed acyclic graph (DAG), meaning that a more specific, child term can have more than one, more general, parent term (i.e., there can be more than one path to a term). Reviews and associated research literature are used to build the pathway diagrams and descriptions. They are also used to annotate the human, mouse, and rat genes to the pathway terms. The rat genes are also functionally annotated using the Gene Ontology (GO). As mentioned, a direct link in the synopsis of an altered pathway to its normal version is provided and within an altered pathway the affected connections/relations are deleted, as necessary. Viewing the altered and normal pathway ‘side-by-side’ provides an instant snapshot of the affected gene-gene relationship(s) and their possible outcomes. Culprit genes are annotated to disease and altered pathway terms and also to the appropriate disease term in the disease ontology (RDO) used at RGD. RGD uses several ontologies for the annotation of genes, QTL, and strains—some are developed by outside sources and in some cases RGD may contribute terms (RDO is the RGD version of the MEDIC disease vocabulary from the Comparative Toxicogenomics Database (CTD)) [15]; others, like PW, are developed at RGD (see for instance [16]).

Resources such as KEGG [8], the Small Molecule Pathway Database (SMPDB) [17], and Reactome [18] offer pathway diagrams, including disease pathways, along with links to gene entries and other pertinent information. However, the portrayal of disease pathways as the collection of altered pathways associated with a particular deviant phenotype, the use of a dedicated pathway ontology to annotate gene products and for use as a navigational tool conferring the ability to ‘travel’ the road of connected pathways, along with the provision of pathway suites and suite networks, are all distinctive features of the RGD Pathway Portal.

## 2 Results and discussion

**Figure 5:**
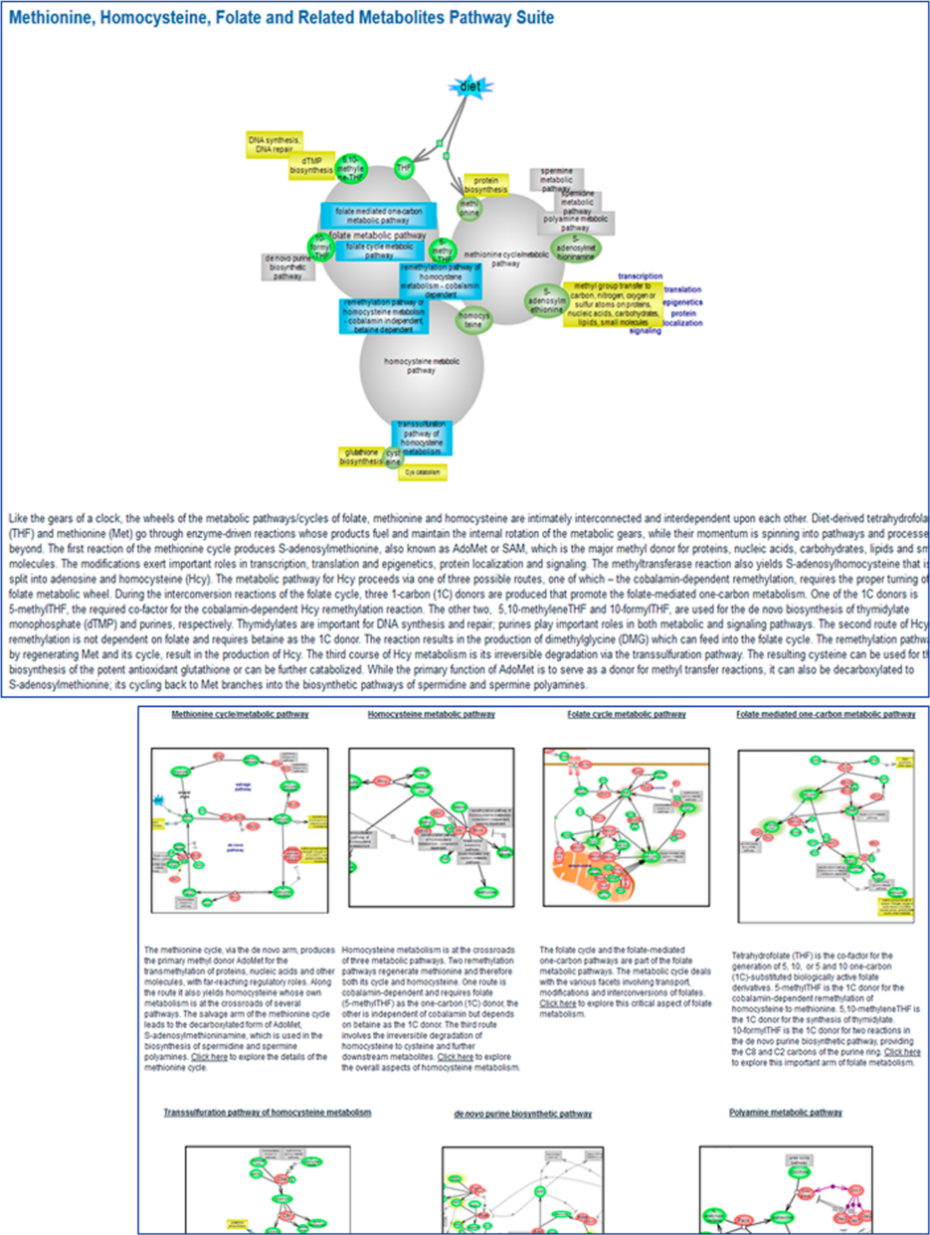
**The Methionine, Homocysteine, Folate and Related Metabolites Pathway Suite.** An overall depiction of the interconnectedness of methionine, homocysteine, and folate metabolic cycles along with other pathways within and/or branching off accompanied by a brief description. Snapshots of the individual pathways within the suites are provided with brief descriptions that link—from the title, image, and description—to the respective interactive pathway diagram page.

The RGD Pathway Portal houses an ever-increasing collection of interactive diagram pages for metabolic, regulatory, signaling, drug, and disease and associated altered pathways along with related pathway suites and suite networks for those pathways that globally revolve around a common concept. Most of the current disease pathway diagrams are pertinent to various cancer types such as colorectal, endometrial, lung, pancreatic, prostate, or renal cancer [see Figure 2]. Diagrams for associated drugs include cisplatin—used in the treatment of several tumors, the anti-estrogen tamoxifen and aromatase inhibitors— used in the treatment of breast cancers, axitinib—used in the treatment of renal cell carcinoma, and gefitinib—used in the treatment of non-small cell lung cancer. As chemicals, including drugs, can exert adverse side effects, at times severe enough to be toxic, response pathway terms and diagrams are used to capture these potentially toxic outcomes and reactions. For instance, the above mentioned drug cisplatin is known to exert severe kidney, neurotoxic, and ototoxic effects—the latter can lead to deafness and is particularly severe in young children. Bisphenol A (BPA) is a compound used in the production of many types of plastics, the lining of food containers, medical and dental devices, and thermal paper. It is also an endocrine-disrupting chemical (EDC) which interacts with several nuclear and hormone receptors but primarily targets estrogen receptors and has been implicated in the etiology of numerous diseases, including cancer. To illustrate the utility of the RGD pathway suite, the BPA response pathway links to estrogen signaling and estrogen signaling links to the biosynthesis of estradiol and both the estrogen signaling and estradiol biosynthesis have links from the anti-estrogen drug pathway (which has links to the individual drug pathways). It is the ‘Estrogen Pathway Suite’ that brings everything together—estrogen signaling and estradiol biosynthesis, the biosynthesis of cholesterol—the precursor of all steroid hormones, and signaling by the sterol regulatory element-binding protein which controls cholesterol and lipid homeostasis, along with the anti-estrogen drug, and the BPA that targets the estrogen receptor. In another group of related pathways, the folate cycle [see Figure 4] and accompanying folate-mediated one-carbon metabolic pathway, intimately connected with the methionine and homocysteine cycles—together interdependent upon each other and each further branching into *de novo* purine biosynthesis, the transsulfuration or the remethylation pathways of homocysteine metabolism, as needed, or polyamine biosynthesis are all members of the ‘Methionine, Homocysteine, Folate and Related Metabolites Pathway Suite’ [Figure 5]. The methionine cycle produces the almost universal methyl donor—S-adenosylmethionione (SAM) used in the methylation of DNA, histones, and other molecules. Aberrant gene methylation is observed in many cancer types. An example of a suite network is the tripartite ‘Balancing Blood Pressure Regulatory Mechanisms Suite Network’ featuring three suites for pathways with a role in mediating an increase or a decrease in blood pressure or both. Several ‘case-studies’ are presented in detail as examples.

## 3 Case studies

### 3.1 Pancreatic cancer pathway

Pancreatic cancer is one of the most aggressive cancer types which despite sustained scientific and clinical efforts continues to have a less than 5% overall 5-year survival rate. It is the fourth leading cause of cancer-related death and both men and women are affected. Pancreatic ductal adenocarcinoma (PDAC) is the predominant form with neoplastic precursor lesions such as pancreatic intraepithelial neoplasia (PanIN) graded stage I to III, converging into PDAC and ultimately invasion and metastasis. A major genetic driver is Kras of the Ras family believed to be an initiator of PDAC and a promoter of its development and progression. Activating mutations are present in >90% of PDAC and can be found as early as PanIN grade I and even in normal pancreas. KRAS activation found in the early stages of pancreatic cancer is followed by inactivation of the cell cycle regulator CDKN2A (95%) also in the earlier stages, and inactivation of TP53 (75%) and SMAD4 (55%) in the later stages. The *CDKN2A* gene codes for two non-identical proteins of which the p16 or p16/Ink4a product is the cyclin-dependent kinase inhibitor and cell cycle regulator and the p14 or p14/Arf product is a p53-activating tumor suppressor. The inactivating mutations in PDAC involve the p16/Ink4a gene product [19]-[21]. However, p14/Arf mutations are associated with other cancer types, and the two protein products act in connected pathways—p53 signaling promotes, as necessary, cell cycle arrest, apoptosis, or senescence and is also a negative regulator of p14/Arf expression [22],[23]. Studies, large-scale genomic studies in particular, indicate that many important cellular pathways are deregulated in pancreatic cancer, those involving the above mentioned genes as well as others [24]-[26] and references therein. These pathways are major players in the regulation of cell growth, differentiation, proliferation, and/or survival, such as the extracellular signal-regulated Raf/Mek/Erk (ERK1/2) pathway downstream of Kras, the Smad-dependent transforming growth factor-beta (TGF-beta) pathway, or the epidermal growth factor/neuregulin (EGF) signaling pathway. Other involved genes are known tumor suppressors such as Tp53 (P53), whose signaling bridges DNA damage response to cell cycle arrest or apoptosis, or important regulators such as Pten in the phosphatidylinositol 3-kinase-Akt (PI3K-Akt) signaling pathway and Cdkn2a in the G1/S transition pathway of the cell cycle. PI3K-Akt may also be downstream of oncogenic Kras in PDAC. Signaling by Hedgehog proteins or hepatocyte growth factor (SF/HGF) play important developmental roles, and both pathways are deregulated in pancreatic cancer. Finally, other involved pathways are integrin-mediated signaling, with important roles in adhesion, and canonical Wnt signaling, with important roles in development. Alterations in components of chromatin modification and remodeling pathways have also been identified. Prominent pathways shown to be altered in the condition are represented in the ‘pancreatic cancer pathway’ diagram page [see Figure 2]. While the substantial number of pathways observed to be affected may be the result of large-scale experimental approaches, it is nonetheless possible that this is a feature particular to pancreatic cancer and as such it may underlie the extreme aggressiveness of this malignancy. The combined effect of these altered pathways can ‘meet’ most if not all the above mentioned ‘hallmarks’ of cancer as put forward by Hanahan and Weinberg [11]. A number of these pathways, particularly those involving Kras, Pten, or EGF, are also deregulated in many other cancer types. Ras proteins were the first oncogenes identified in human cancers, primarily Kras with frequent activating mutations and, to a much lesser extent, mutations in the closely related Hras or Nras genes. Kras confers stem-like properties on certain cell types, Kras deletion leads to death during embryogenesis in mice, and it is in pancreatic cancer that the activating Kras mutations are the most frequent [27]. miRNAs, which primarily inhibit the translation of target genes, are aberrantly expressed in pancreatic cancer as well as other cancer types. Many miRNAs are shown to be both up- and down-regulated in pancreatic cancer resulting in multiple effects such as chemoresistance, proliferation, survival, invasion, and metastasis [12]. Early detection of pancreatic cancer has proven difficult. Aberrant DNA methylation and other epigenetic alterations are observed in many cancer types, including pancreatic cancer. Epigenetic markers may provide a means for earlier detection of pancreatic cancer [28]. Pancreatic cancer may be an extreme case but it is nonetheless intriguing why so many mutations and associated altered pathways converge on this organ and its malignancy and why they do display a preferential as well as a temporal pattern.

### 3.2 Prostate cancer pathway

Unlike pancreatic cancer with its plethora of altered pathways, in the case of prostate cancer, the main culprit is the androgen receptor (AR) of the androgen signaling pathway. A deregulated phosphatidylinositol 3-kinase-Akt pathway also contributes with loss or inactivating mutations in the Pten regulator and, to a lesser extent, amplification or activating mutations of the catalytic subunit Pik3ca. Deletions at the p53 locus in the p53 pathway have also been seen in prostate cancer samples [29]. Normal androgen signaling plays an essential role in the development and maintenance of the male phenotype and reproductive functions and in a range of other processes. The steroid hormone AR belongs to the nuclear receptor family. Once activated by hormone binding—testosterone or its more potent metabolite dihydrotestosterone (DHT), AR translocates to the nucleus to regulate the expression of its target genes. In the altered pathway leading to prostate cancer, the androgen receptor displays an intriguing altered and mutational behavior, mostly treatment-induced. Reduction of testosterone levels leads to changes in gene copy number and also in increased transcription of the receptor. Thus, trace amounts of the ligand are enough to set its signaling pathway in motion. Complete elimination of plasma testosterone initially abrogates receptor activity, followed in time by various mechanisms which result in ligand-independent androgen receptor signaling. Increased phosphorylation of the receptor, expression of splice variants that do not have the ligand binding domain, mutations that allow the receptor to bind to and be activated by non-traditional ligands including antagonists, and altered expression of coregulators, steroid biosynthetic enzymes, and/or the receptor transcriptional program all contribute to testosterone-independent receptor activity [30]-[34]. Alterations in the NCOA2 coactivator and NCOR2 corepressor and in chromatin regulatory elements have been reported [29]. Steroid hormones share a core biosynthetic pathway that branches into the individual steroid biosynthesis in given tissues via tissue-specific expression of terminal enzymes; in prostate cancer, changes in enzyme expression circumvent the initial testosterone depletion [31]. Gene fusion between androgen receptor-regulated genes and members of the E26 transformation-specific (ETS) family of oncogenic transcription factors is a signature feature found in ~50% of cases. Fusion between the *Tmprss2* gene and the *Erg* member of the ETS family is common in prostate cancer. The gene rearrangements have been mostly noticed in primary prostate cancer while the alterations/mutations of the receptor have been associated with the treatment-resistant, metastatic prostate tumors. The PI3K-Akt and p53 alterations are observed in both primary and metastatic prostate cancer. Mutations in a number of other elements are being unraveled and await further investigation with respect to their role in the initiation and/or progression of prostate tumors [29],[32],[33]. In the altered androgen signaling and prostate cancer pathways, the androgen receptor becomes a ligand-independent or antagonist-induced, constitutively active transcription factor.

### 3.3 Renal cell cancer pathway

**Figure 6:**
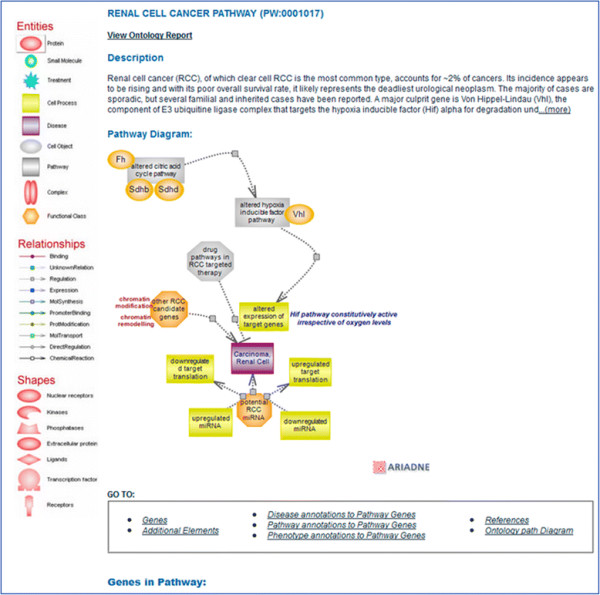
**Renal cell cancer pathway.** The interactive pathway diagram page for the ‘renal cell cancer pathway’ with the associated altered pathways and culprit genes. The icons for the miRNAs with potential implications, other candidate genes, and drug pathways, link to pages with related information and links within.

Like prostate cancer, renal cell cancer (RCC), of which the clear cell RCC (ccRCC) is the most common type, results from a relatively small number of alterations in known pathways [Figure 6]. The main contributor in this case is an altered hypoxia-inducible factor (HIF) pathway where mutations in von Hippel-Lindau (VHL) protein render the pathway constitutively active and are found in ~90% of RCC tumors [35]. The HIF pathway is considered a master regulator of oxygen homeostasis. HIF is a heterodimer composed of a constitutively expressed subunit beta also known as the aryl-hydrocarbon receptor (Arnt) with roles in several transcription pathways and the alpha subunit whose protein levels are highly regulated by oxygen and also in an oxygen-independent manner. Both subunits are members of the basic helix-loop-helix (bHLH) and PER-ARNT-SIM (PAS) domain-containing family of transcription factors. Under normoxic conditions, the HIF proteins are subject to proline and asparagine hydroxylation carried out by enzymes that are oxygen-dependent and require iron and 2-oxoglutarate for activity [36]. The modified prolines are recognized by VHL which targets HIF for proteasomal degradation; the modified asparagine blocks the interaction of HIF with transcriptional activators [see Figure 3A]. There are three HIF alpha isoforms of which alpha 1 and 2 are thought to be the primary VHL targets. Both isoforms are believed to play a role in RCC, but it is the alpha 2 protein that appears to have the predominant role in tumorigenesis [37]. The two isoforms have common and also distinct targets with angiogenic genes being under the control of both isoforms while genes involved in cell proliferation, dedifferentiation, and invasion are under the control of alpha 2 [38]. Specific mutations cataloged for the *VHL* gene are thought to underlie distinct morbidity and mortality phenotypes. Mutations in components of the citric acid cycle (tricarboxylic acid or TCA cycle) create a pseudohypoxic environment that impacts on Hif modification, stabilizes the protein, and renders the pathway active, even when the VHL protein may be wild-type [39]. In addition, large-scale sequencing experiments have identified genetic changes in a number of genes whose protein products have epigenetic roles such as histone methylation and demethylation in ~15% of RCC cases. Also, truncating mutations in the *PBRM1* gene known as BAF180 (a component of a chromatin remodeling complex) were found in ~41% of cases [37]. The exact role these mutations have in the context of RCC remains to be established. It is possible that they affect, in some fashion, the transcriptional activity of HIF. It is worth mentioning that, in the case of the alpha 1 isoform, studies show binding and regulation of expression of several Jumonji domain-containing histone lysine demethylases [40]. *VHL* is a tumor suppressor gene first identified in patients with VHL disease—a rare hereditary cancer syndrome that exhibits vascularized tumors of the retina and other tissues. RCC, however, is primarily a sporadic condition, where bi-allelic inactivation of VHL due to chromosomal loss, mutations, or hypermethylation accounts for much of its incidence [41],[42].

In RCC, as in the case of prostate cancer with altered androgen signaling, HIF has become an unregulated, condition-independent, constitutively active transcription factor. However, unlike the androgen receptor pathway with its rather distinct expression and functional range, the HIF pathway is ubiquitous, yet its alteration is localized. VHL, the dominant contributor to RCC, is rarely mutated in other sporadic tumors.

### 3.4 Drug and drug/compound responses—axitinib, cisplatin, and bisphenol A

**Figure 7:**
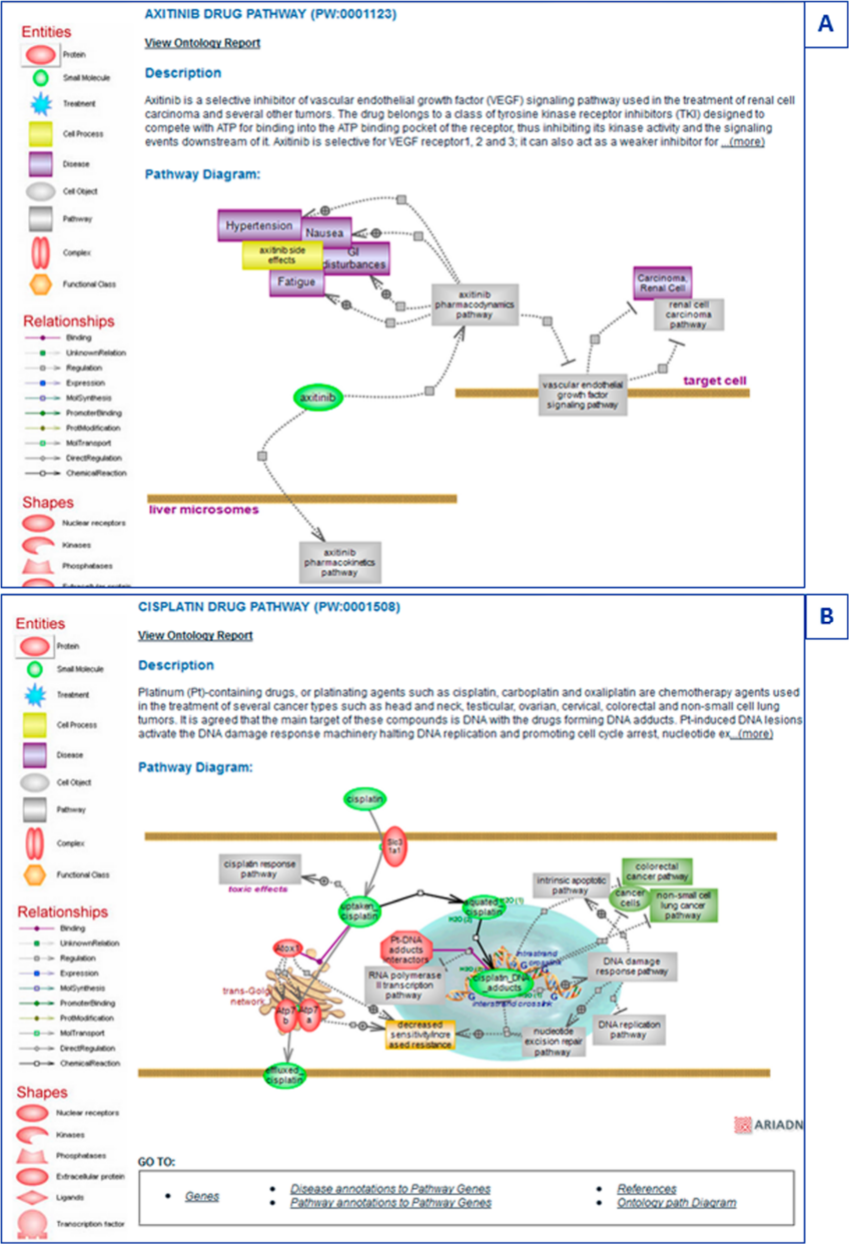
**Drug pathways. (A)** The overall drug pathway for axitinib with associated pharmacokinetics and pharmacodynamics pathways and targeted pathways. **(B)** The interactive pathway diagram for the drug cisplatin.

Several drugs have been developed for the treatment of RCC (see ‘Renal cell cancer pathway’); their targets include the vascular endothelial growth factor (VEGF) and its receptors downstream of Hif-mediated transcription or the mTOR pathway which regulates HIF translation [35]. Most drugs target the VGEF receptors and among them is axitinib—a drug selective for VEGF receptors 1, 2, and 3 that competes with ATP for binding into the ATP binding pocket of the receptor. The drug pathway presents an overall depiction of axitinib action which includes the pharmacokinetics and pharmacodynamics pathways, targeted pathways, and side effects [Figure 7A]. In many cases, the pharmacokinetics arm of the drug pathway shows the human genes as many experiments are carried out in human liver microsomes and the enzymes involved in drug metabolism have, in many cases, broad substrate specificity and/or have not been well conserved throughout evolution.

**Figure 8:**
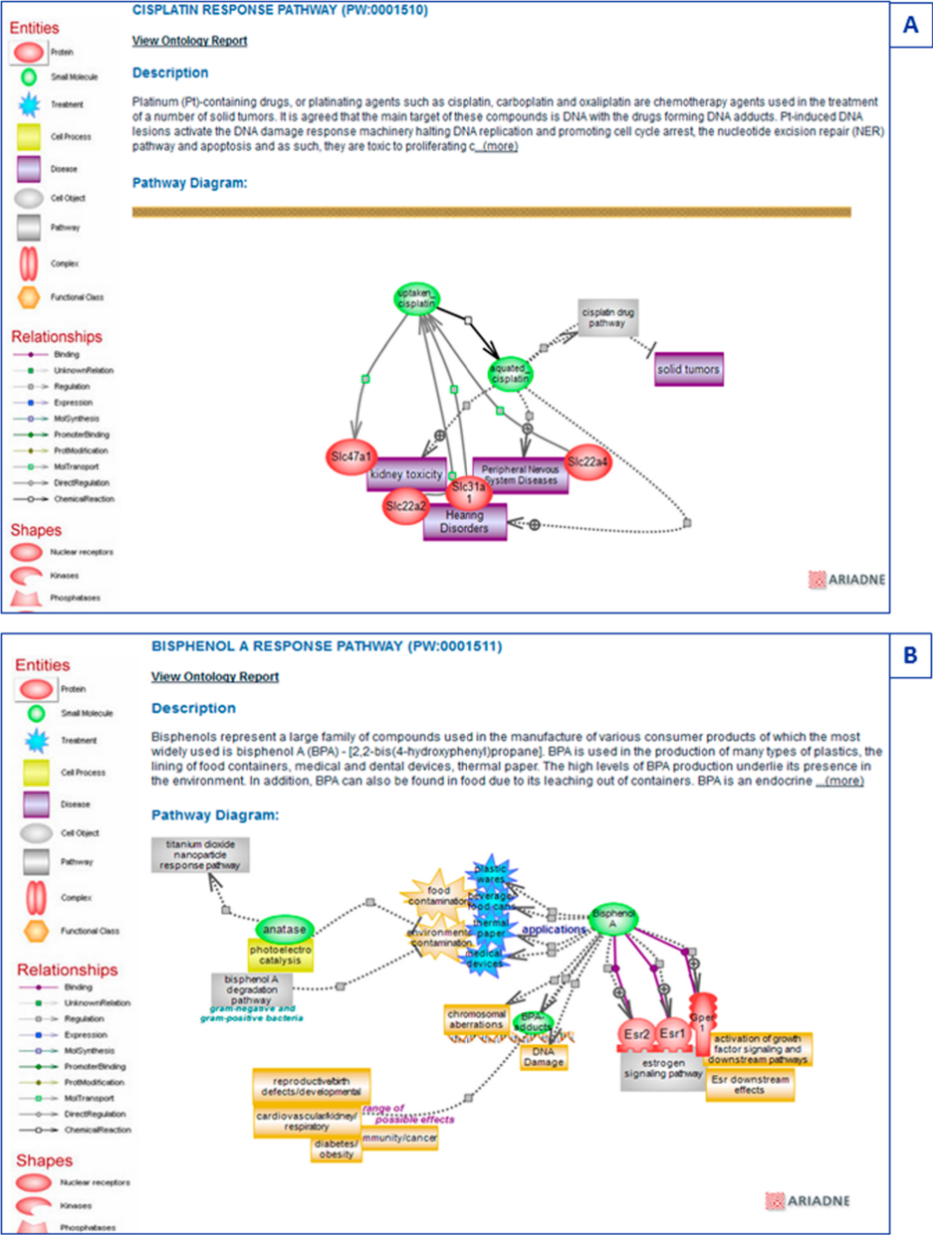
**Drug response pathways. (A)** The diagram page for the cisplatin response pathway showing the toxic effects of the drug and the genes that may be responsible for its accumulation. **(B)** The diagram page for the bisphenol A (BPA) response pathway showing its uses, targets, and effects.

Unlike axitinib, which is selective for a particular target and is primarily used in the treatment of RCC, cisplatin is a drug used in the treatment of numerous cancer types. It is a platinating agent whose main target is agreed to be DNA where cisplatin and other platinating drugs form various DNA adducts. The DNA lesions activate the DNA damage response (DDR) system which, among a number of responses, can promote apoptosis and as such is toxic to proliferating cancer cells. The pharmacokinetics and pharmacodynamics of cisplatin are presented in a single diagram as the drug does not have a specific target [Figure 7B]. Cisplatin is the most potent of the platinating agents and is also the most toxic [43]. Neurotoxic, ototoxic, and renal effects have been reported, with the ototoxic effect being the most severe. The hearing loss associated with cisplatin treatment can lead to deafness and is particularly severe in children. The incidence of ototoxicity is reported to be 23%–50% for adults and greater than 50% for children. Cisplatin exerts similar effects in non-cancer cells as it does in cancer cells. Increased exposure/accumulation, changes in transporter levels and/or DNA repair genes, and epigenetic or regulatory differences may render certain cells more prone to undergo apoptosis, adding to the observed toxic effects. The cisplatin response pathway shows these effects and lists the transporters that are thought to be responsible in various tissues [Figure 8A].

Like cisplatin, BPA can form DNA adducts, but its primary targets are nuclear and hormone receptors, mainly the estrogen receptors. BPA is used in the production of many types of plastics, the lining of food containers, medical and dental devices, and thermal paper. The high levels of BPA production are responsible for its widespread presence in the environment. In addition, BPA can also be found in food due to its leaching out of containers. BPA binds the ESR1 and ESR2 nuclear estrogen receptors and also the G protein-coupled estrogen receptor GPER. BPA can induce rapid activation of the Erk1/2 pathway via GPER in breast cancer; it may affect various immune responses and has been implicated in the etiology of many diseases and disorders [44] [Figure 8B]. Most compounds and drugs exert negative effects; the distinction between adverse effects and toxicity is one of response, i.e., the relative steepness of the response curve. As in the altered and disease pathways, the diagrams attempt to capture the molecular underpinnings of drug metabolism and of drug-target interactions or the implications of drug and/or chemical toxicity

### 3.5 Estrogen pathway suite

**Figure 9:**
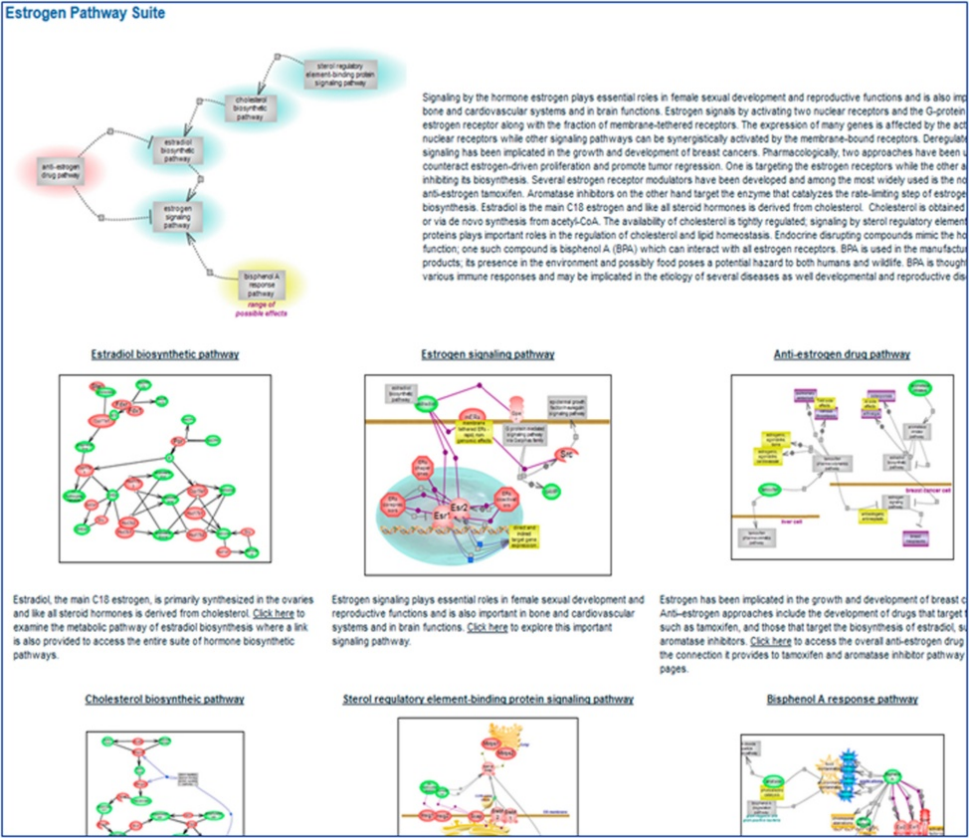
**The estrogen pathway suite.** The pathways revolving around estrogen are shown with their connections and a brief description. The individual pathways are shown as snapshots with links to the respective diagram page from the title, image, and accompanying description.

Estrogen signaling is essential for female sexual development and reproductive functions and plays important roles in bone and cardiovascular system function and in brain function [see Figure 9]. Estrogen activates two nuclear receptors, ESR1 and ESR2, the G protein-coupled estrogen receptor GPER, and the fraction of membrane-tethered ESR receptors. Estrogen signaling affects the expression of many genes. Deregulation of estrogen signaling is implicated in the etiology of breast cancer, and signaling is constitutively active in more than 50% of cases. The estrogen signaling pathway is a target for the development of anti-estrogen drugs. Pharmacologically, two approaches are being used to counteract estrogen-driven proliferation and promote tumor regression. One is targeting the receptors, and tamoxifen is an example of a selective estrogen receptor modulator (SERM). Tamoxifen binds competitively to the estrogen receptors acting as an antagonist in breast cancer cells and as a partial agonist in bone and cardiovascular systems. The pharmacokinetics of tamoxifen are complex with both primary and secondary metabolites being formed whose potencies are greater than that of the parent compound. The metabolites are inactivated via glucuronidation and sulfation, the former is the main route. The other pharmacological approach is targeting the synthesis of estrogen. Estradiol, the main C18 estrogen, is primarily synthesized in the ovaries and, like all steroid hormones, is derived from cholesterol. The steroid hormones biosynthetic pathway consists of a core of common reactions that branches out into the production of individual hormones through the action of end terminal enzymes whose expression is confined to particular tissues. In the case of estrogens—estradiol (E2) and estrone (E1), this involves CYP19A1, a cytochrome P450 member, known as aromatase. Three generations of aromatase inhibitors have been developed, with the third generation currently being used. The inhibitors are further classified as type I or type II depending on whether the inhibition is irreversible or reversible. Cholesterol, the precursor of steroid hormones and an essential component of cell membranes and lipid rafts, is under the control of the sterol regulatory element-binding protein (SREBP) signaling pathway. The SREBF (SREBP) proteins represent a subfamily of basic helix-loop-helix leucine zipper transcription factors whose signaling regulates cholesterol and lipid homeostasis and, in turn, is regulated by the levels of lipids. The nuclear and G protein-coupled estrogen receptors are also targets of BPA whose actions have been implicated in the etiology of many diseases, disorders, and affected processes. The various pathways revolving around estrogen are brought together within the ‘Estrogen Pathway Suite’ to provide an instant glimpse of their associations while individual examination provides a tool for studying and exploring the underlying molecular events.

## 4 Conclusions and future developments

A disease pathway brings together associated altered pathways and culprit genes within, along with deregulated miRNAs and other putative candidate genes, to provide a unique view of the possible molecular mechanisms underlying the condition. Normal processes are hijacked, harnessed, and modulated by tumor cells to serve their proliferative and invasive needs, as posited by Hanahan and Weinberg [11]. The case studies presented here centered on malignancies and featured a number of special examples: the extreme case of pancreatic cancer with its many altered pathways; prostate cancer with few contributors and whose main culprit, despite its intriguing therapy-induced resistance response, can be rationalized by virtue of specific expression and function; and renal cell cancer, also with few contributors, but whose main culprit cannot be readily explained by virtue of expression and function alone. Despite the identity of altered pathways being different, constitutive signaling as well as deregulated expression of coding and non-coding genes, activation of oncogenes, and silencing of tumor suppressors are shared features. However, there are also distinctive features and they raise questions. For instance, many of the pathways altered in pancreatic cancer are known to be oncogenic in other cancer types—as an example, *TP53* is the most commonly mutated gene in cancer. However, it is *KRAS* that has the highest mutation incidence in pancreatic cancer—twice its occurrence in cancer of the colon or small intestine, followed in decreasing order by endometrium and lung cancers and much lower in neoplasms of other tissues. Why is *KRAS* preferentially mutated in pancreatic cancer and why does this member of Ras have the highest overall mutagenic propensities relative to the other two, closely related Ras members, HRAS and NRAS? Differential codon usage in *KRAS* might underlie its higher mutagenic rate than its Ras cousins and distinct mutations may confer distinct functional behaviors upon the mutant proteins and prompt different outcomes, but they do not explain what triggers the frequency of mutations in and their association with different tissues [27]. In a similar vein, one may ask why somatic mutations in the VHL protein are primarily associated with renal cell cancer or why diminishing levels of testosterone prompt the kind of mutations that render the androgen receptor ligand-independent.

Interestingly, the pancreatic acinar cells and the most abundant cell type, under certain conditions such as injury and accompanying inflammation, can acquire plastic capabilities [45]. Findings suggest that acinar cells may be the cells of origin for PanIN lesions and renal ductal adenocarcinoma. These findings await further investigation to be firmly established. However, one is tempted to wonder whether the proneness of *KRAS* to mutate may in some fashion be aided or activated within a challenged pancreatic tissue milieu, perhaps further promoting its plasticity and the instantiation of mutations in other genes. Likewise, do the unique features of the filtering system the kidney represents make it more vulnerable to unwanted hypoxic responses which would be easy to ‘satisfy’, by just silencing VHL? Are the reproductive tissues more likely to be affected by infection, and does a yet to be identified infectious agent confer upon the androgen receptor its features, reminiscent of acquired resistance?

The provision of diagrams offers a means to quickly visualize the individual aspects of given pathways and, in the case of disease pathways, to inspect the underlying altered pathways, how their unfolding differs from the regular ones, what features are shared, and which ones may be unique. Having access to a large collection of disease and associated altered pathways enables the user to quickly inspect, compare, and identify aspects that may be unique or aspects that may be intriguing. As such, it can prompt asking new questions or redefining previous ones, lead to the search for new or revised venues of inquiry, and overall help further the efforts aimed at deciphering the mechanisms that determine the initiation and progression of disease. Future work will focus on expanding the repertoire of published diagrams for disease and altered pathways and associated drug and response pathways. Also, pathways underlying epigenetics and transcriptional programs, which are at the heart of differential gene expression in which genes get expressed and/or spliced at certain times in certain locations, likely play a role in the issues raised above. In addition, new pipelines and new features/entries of individual diagrams, such as those for genes with non-synonymous variants in the sequenced rat strains and genes and variant structural models, are scheduled to be implemented. The overall increase of the interactive diagram collection and associated pathway annotations for the rat, human, and mouse genes represent an ongoing process in the multifaceted Pathway Portal project.

## 5 Methods

The Pathway Ontology is being developed using the OBO ontology editor, developed by the Gene Ontology Consortium [46]. The pathway diagrams are being built using Pathway Studio software, version 9, from Elsevier [47]. The pathway diagram pages are made using a pathway curation tool web application developed at RGD [48]. The methodology for building the pathway pipelines is detailed in Petri et al. [1].

## Competing interests

The authors declare that they have no competing interests.

## Authors’ contributions

VP wrote the manuscript, originated the PW ontology, composed most of the PW diagrams, and assisted with the portal design, diagram publications, and pathway pipeline construction. GTH composed some of the PW diagrams, assisted with the diagram publications, and reviewed the manuscript. MT assisted with the PW uploads, ftp file placement, and pipeline implementation. JRS assisted with the diagram publications. SJFL and SJW reviewed the manuscript. RN is part of the RGD team. JDP supervised the pipeline construction. MS supervised the PW portal and pipeline projects. MRD, EAW, and HJJ cosupervised the RGD projects. All authors read and approved the final manuscript.
